# Transjugular Patent Foramen Ovale Closure in the Setting of Inferior Vena Cava Interruption

**DOI:** 10.1016/j.jaccas.2026.110227

**Published:** 2026-09-05

**Authors:** John P. Birrane, Marin Roman, Gregory Offiah, Aine O’Connor, Ross T. Murphy, Sandra Quinn, Kevin P. Walsh, Andrew O. Maree

**Affiliations:** a Department of Cardiology, St James’s Hospital, Dublin, Ireland; b The Institute of Cardiovascular Science, Trinity College Dublin, Dublin, Ireland; c Children’s Health Ireland at Crumlin, Dublin, Ireland

**Keywords:** cryptogenic stroke, IVC interruption, paradoxical embolism, patent foramen ovale closure, transjugular access

## Abstract

**Background:**

Percutaneous patent foramen ovale (PFO) closure typically uses femoral venous access. Congenital or acquired inferior vena cava (IVC) interruption precludes this route and presents a significant technical challenge.

**Case Summary:**

A 54-year-old man was referred for PFO closure following cryptogenic embolic stroke. Femoral access failed; intraprocedural venography confirmed IVC interruption. The case was reattempted via the right internal jugular vein. A 30-mm PFO closure device was deployed using a preshaped transcatheter aortic valve replacement guidewire anchored in the left ventricle for support.

**Review Summary:**

Fifteen published cases detail PFO closure via transjugular, transhepatic, and upper-limb access. The transjugular approach requires specific strategies to overcome the unfavorable crossing angle, including preformed or steerable catheters and distal wire anchoring in the pulmonary veins, left atrium, or left ventricle.

**Take-Home Messages:**

Congenital or acquired IVC interruption necessitates alternative access strategies for percutaneous PFO closure. While technically demanding, transjugular and transhepatic routes are feasible alternatives.

## Visual Summary

**Figure:**
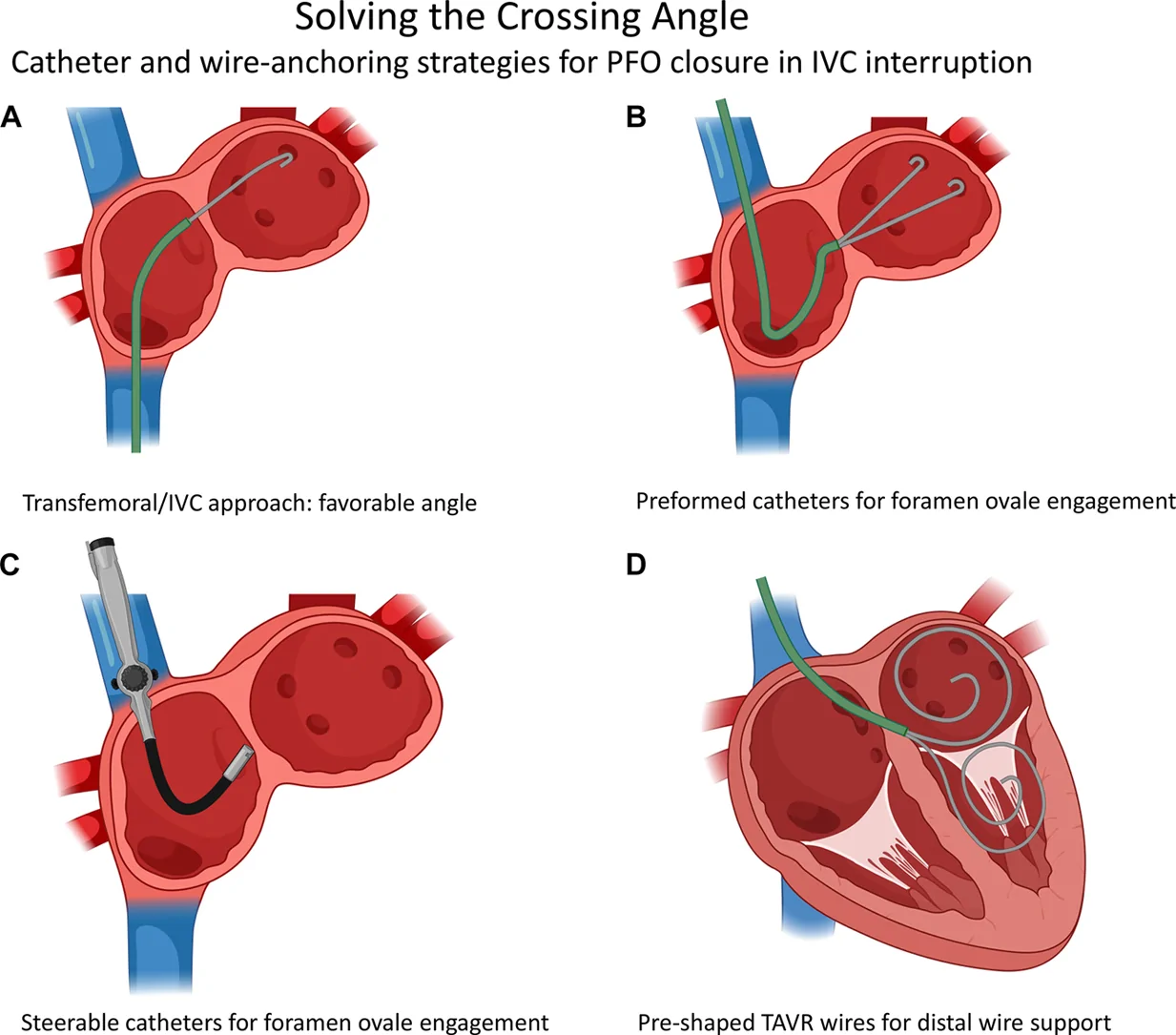

Transcatheter closure of a patent foramen ovale (PFO) reduces recurrent stroke risk beyond medical therapy alone in patients who suffered a cryptogenic stroke and has a high-risk interatrial septal anatomy. Femoral venous access is the standard approach and provides reliable access to the interatrial septum (IAS) in most cases.

## History of Presentation

A 54-year-old male presented to a hospital with acute-onset dysarthria and expressive dysphasia. He had no other neurological deficits. An urgent computed tomography (CT) brain showed a possible left basal ganglia infarct, and he was admitted for further investigation.

## Past Medical History

Past medical history was significant for hypertension, obstructive sleep apnea, and surgical intervention for a perforated duodenal ulcer 23 years earlier.

## Investigations

Magnetic resonance imaging brain confirmed left basal ganglia infarction. CT angiography of the aortic arch and cerebral vessels was unremarkable.

Inpatient cardiac monitoring showed sinus rhythm. A transesophageal echocardiogram (TEE) revealed a bicuspid, nonstenotic aortic valve. An agitated saline bubble study demonstrated a PFO with a significant right-to-left shunt (Figure 1).

**Figure 1:**
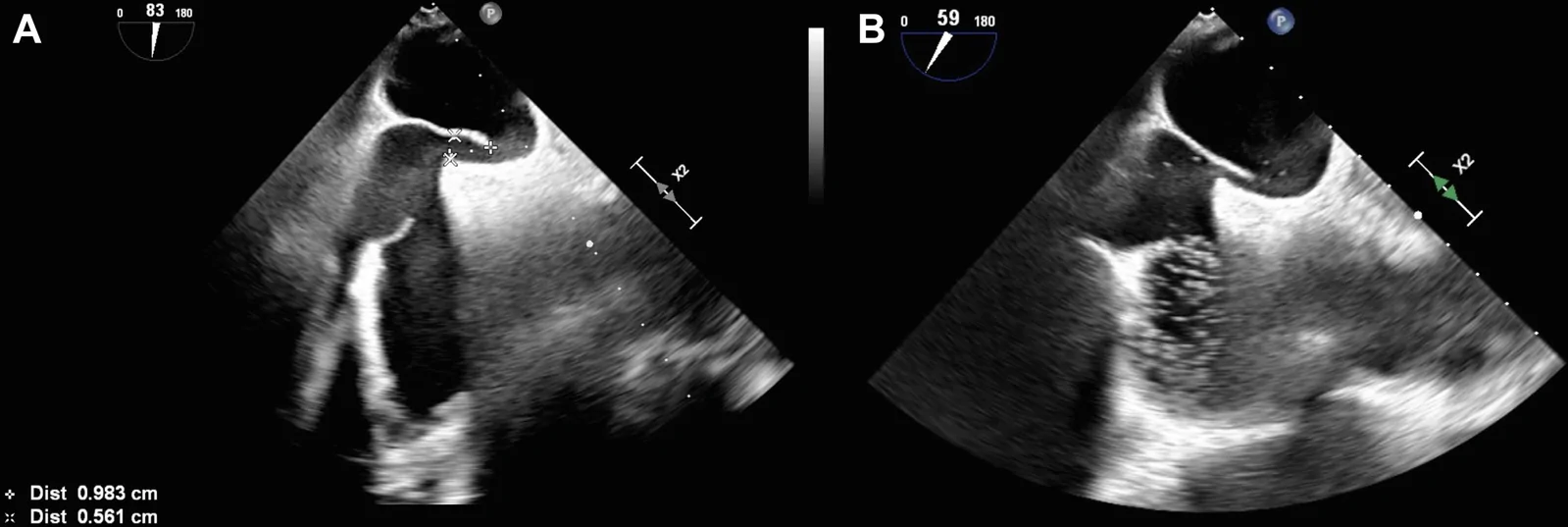
Tunneled Patent Foramen Ovale With Right-to-Left Shunt on TEE Transesophageal echocardiography demonstrated a tunneled patent foramen ovale of almost 10 mm with clear separation of primum and secundum septae (A). A shunt was demonstrated with an agitated saline bubble study, with bubbles crossing the interatrial septum from right to left within 2 cardiac cycles (B).

A thrombophilia screen, testing for Factor V Leiden, cardiolipin antibodies, and beta-2 glycoprotein antibodies, was negative.

An implantable loop recorder showed no atrial fibrillation over 12 months. A Risk of paradoxical embolism score of 5 was calculated, indicating an estimated PFO-attributable risk of approximately 34%.

## Management

The patient’s case was discussed at our heart team meeting, and percutaneous PFO closure was recommended.

The procedure was attempted under general anesthesia with intraprocedural TEE guidance. A 6-F Cook sheath was sited in the right common femoral vein with ultrasound guidance. Despite appropriate venous back-bleeding, attempts to advance a wire to the inferior vena cava (IVC) were unsuccessful. A further attempt was made via the left common femoral vein but was unsuccessful. A left femoral venogram revealed anomalous venous return to the right atrium through a system of collateral veins (Figure 2, Video 1). Given the anatomical uncertainty and inability to cross the collateral system, the procedure was abandoned.

**Figure 2:**
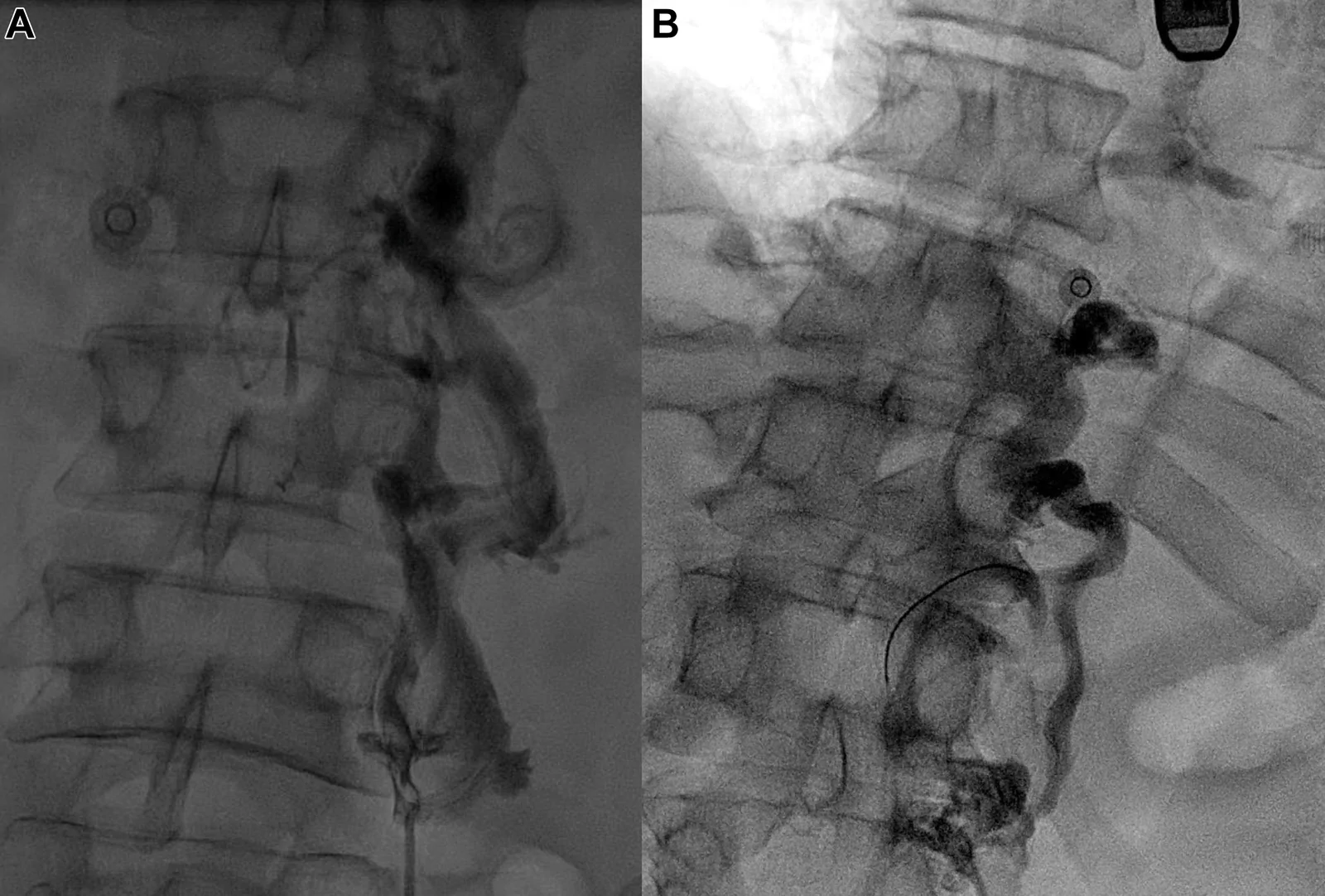
Left Femoral Venography Demonstrating Collateral Venous Return in Inferior Vena Cava Interruption A venogram was carried out through a 6-F Cook sheath within the left common femoral vein (A). It demonstrated an extensive network of venous collaterals in place of a normal iliocaval venous anatomy with reformation of the inferior vena cava for a short segment below the right atrium (B).

A CT venogram confirmed IVC interruption with caudal venous return occurring via lumbar, azygous, and hemiazygos collateral vessels. Venous imaging could not differentiate congenital from acquired etiology.

Following heart team discussion and review of the published literature for PFO closure through alternative access, the case was reattempted via the right internal jugular vein. The patient’s mobile IAS and PFO tunnel length of almost 10 mm led to a consensual decision to use a 30-mm Amplatzer Talisman PFO occluder (Abbott) to cover the defect. An 8-F sheath was inserted under fluoroscopic guidance. Systemic anticoagulation was initiated with heparin, targeting an activated clotting time of >250 seconds.

Step 1: The IAS was crossed from the right atrium using a 5-F AL1 diagnostic catheter, and a 0.035 × 180-cm J-Tip Terumo wire was initially delivered to the left upper pulmonary vein (Figure 3). The angle was unfavorable and provided inadequate support to advance the AL1 diagnostic catheter into the left atrium (LA); therefore, the wire was redirected into the left lower pulmonary vein.

**Figure 3:**
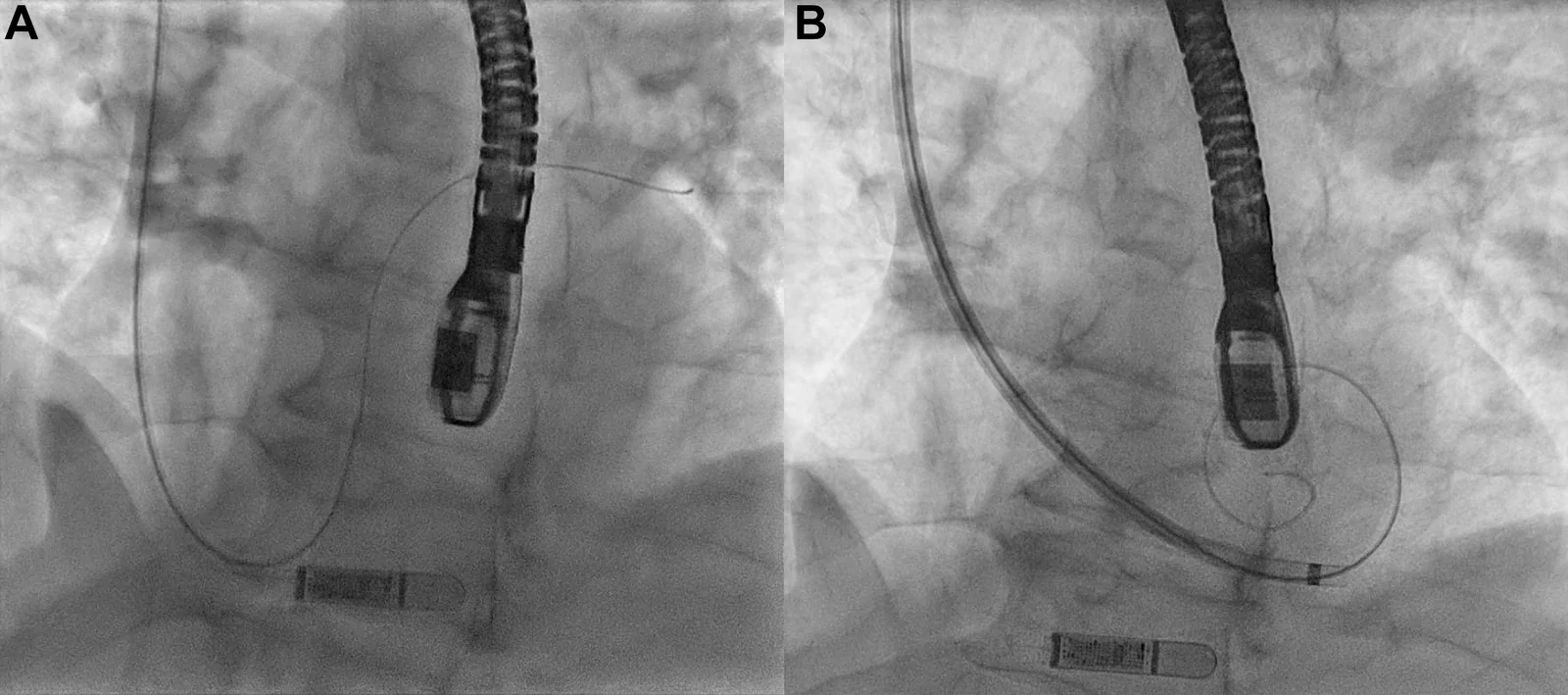
Wire Anchoring Strategies for Septal Crossing From Transjugular Access Initial attempts to cross the patent foramen ovale from the transjugular position involved anchoring a J-tipped Terumo wire in the left upper pulmonary vein via a 5-F AL1 diagnostic catheter (A). This failed to provide adequate support or a favorable angle for crossing. A preshaped transcatheter aortic valve replacement wire was then delivered into the left ventricle, providing adequate support to advance the 8-F Amplatzer Talisman sheath into the left atrium (B).

Step 2: The Terumo wire was exchanged for an Abbott extra-stiff 260-cm guidewire. We attempted to deliver a 6-F Multipurpose (MPA)1 90-cm guide catheter over the guidewire, but it would not cross the septum. A 5-F 110-cm MPA1 diagnostic catheter was inserted over the stiff wire through a Guardian II hemostasis valve (Teleflex) on the back of the guide catheter, and this facilitated successful transition of the MPA1 guide sheath into the left lower pulmonary vein.

Step 3: The MPA1 5-F diagnostic catheter was withdrawn, and a small preshaped transcatheter aortic valve replacement (TAVR) guidewire (Safari, Boston Scientific) was advanced through the 6-F MPA1 sheath, where it prolapsed and assumed a stable position in the left ventricle (Figure 3). This provided excellent support for sheath exchange for an 8-F Amplatzer Talisman sheath and dilator (Abbott). A 30-mm Amplatzer Talisman PFO occluder was successfully deployed across the septum under TEE guidance. The final echocardiographic and fluoroscopic assessments demonstrated excellent alignment and complete septal occlusion (Figure 4, Video 2).

**Figure 4:**
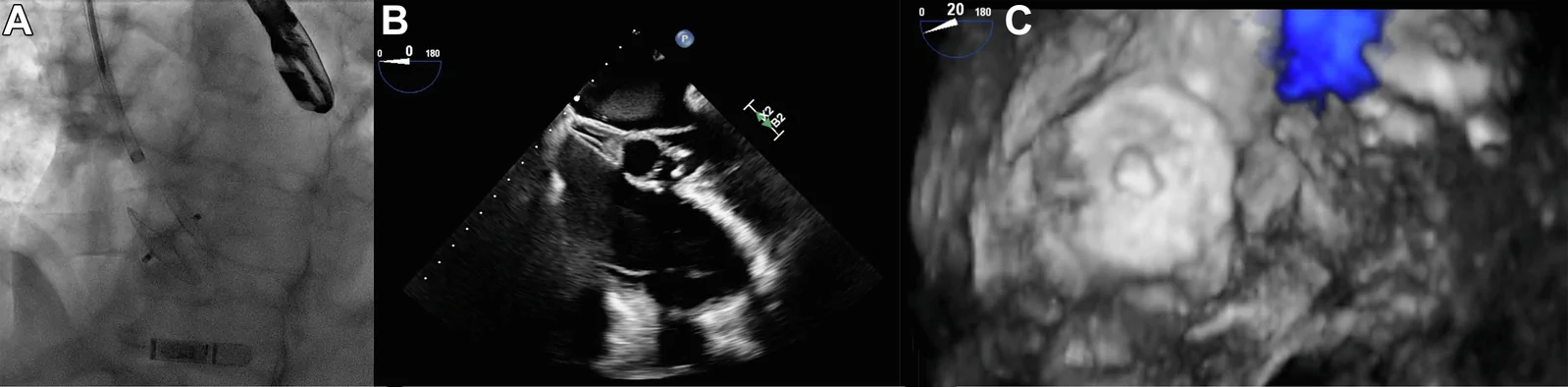
Final Assessment Following Deployment of the 30-mm Amplatzer Talisman Patent Foramen Ovale Occluder Fluoroscopy demonstrates good device alignment across the interatrial septum with both discs well apposed (A). Two-dimensional transesophageal echocardiography confirms appropriate disc position straddling the septum with no residual shunt on color Doppler (B). The three-dimensional transesophageal echocardiography en face view from the left atrium shows the device flat against the septum with no impingement on surrounding structures (C).

**Visual Summary:**
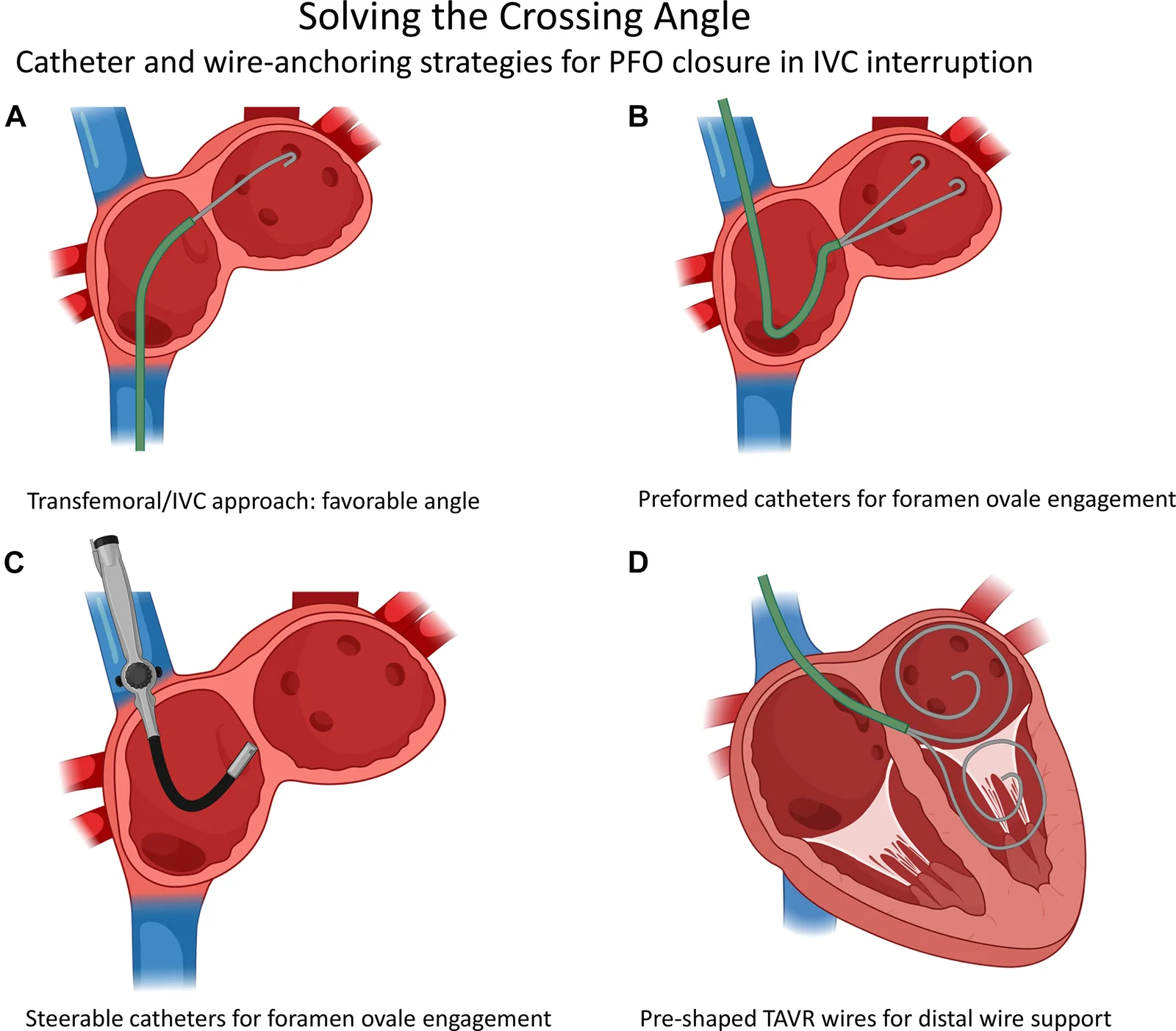
Solving the Crossing Angle: Access and Anchoring Strategies for Transjugular PFO Closure With IVC Interruption Percutaneous PFO closure in the setting of IVC interruption requires an alternative venous access. From a conventional transfemoral or transhepatic approach, the angle between the IVC and the foramen ovale is favorable for septal crossing (A). When access is obtained via transjugular or upper limb venous approaches, the more perpendicular angle between the superior vena cava and the foramen ovale can make engagement and delivery technically challenging (B to D). A range of techniques have been reported to address this. Preformed left coronary catheters can engage the foramen ovale, with subsequent wire anchoring in the left upper or lower pulmonary vein providing support for sheath delivery (B). Steerable catheters offer operator-controlled deflection where preformed catheters do not provide adequate engagement (C). Preshaped TAVR guidewires positioned in the left atrium or left ventricle represent an additional form of wire support (D). The relative effectiveness of these techniques is not established; reported cases describe them used alone or in combination. Created in BioRender. IVC = inferior vena cava; PFO = patent foramen ovale; TAVR = transcatheter aortic valve replacement.

## Outcome

The patient made an uneventful recovery, and he was discharged the following day. He will be followed up with repeat echocardiography as an outpatient.

## Literature Review

Alternative access strategies for PFO closure are required when femoral access is not feasible. IVC interruption independently raises venous thromboembolic risk, roughly 10-fold in a recent multicenter registry, and is strongly associated with thrombophilia.^1^ Whether this translates into greater risk of paradoxical embolism through a PFO is not established, though collateral venous return via the azygos system provides a plausible route by which thrombus can still reach the right atrium.

A PubMed and Embase search (February 2026) using the terms “patent foramen ovale,” “inferior vena cava,” and “interruption” identified case reports of PFO closure with an alternative access. Priority was given to publications that focused on procedural approaches and technical outcomes. Reference lists were screened for additional cases.

### Access approach to PFO closure in patients with IVC interruption

Fifteen case reports of PFO closure in patients with IVC interruption were found (Table 1).^2^, ^3^, ^4^, ^5^, ^6^, ^7^, ^8^, ^9^, ^10^, ^11^, ^12^, ^13^ The mean patient age was 54.9 years (range: 24-78 years), with a male predominance (60%). Acquired occlusive pathologies such as thrombosis, surgical ligation, and thrombosed indwelling IVC filters were the primary cause of venous obstruction (n = 11), while congenital IVC interruption or atresia (n = 4) accounted for the remaining cases.

**Table 1:** Published Cases of PFO Closure in Patients With Inferior Vena Cava Interruption or Occlusion

First Author	Age (y), Sex	IVC Pathology	Access Route	Support Strategy	Procedural Technique	Device (Sheath)
Soodi et al (2024)^2^	60, M	Congenital IVC interruption	Transhepatic (right hepatic vein)	Direct trajectory (transhepatic)	MP catheter across PFO; transhepatic tract coil-embolized for hemostasis	Cardioform 30 mm (NR)
Özdemir et al (2023)^3^	45, F	Acquired IVC compression by uterine myoma with bilateral iliac thrombus	Right internal jugular	Distal wire anchoring in pulmonary vein with coronary balloon	EBU catheter; 5.0 × 15-mm coronary balloon over 0.014-inch wire in pulmonary vein as anchor to deliver catheter across septum; stiff curved wire exchanged for sheath delivery	Amplatzer PFO occluder 25 mm (8-F)
He et al (2022)^4^	42, M	Acquired IVC thrombosis with thrombosed IVC filter	Right internal jugular	Distal wire anchoring in pulmonary vein with escalation to wire anchoring in LA	EBU 3.5 mm guide; 180-cm 0.025-inch coiled-tip stainless-steel wire looped in LA	Amplatzer PFO occluder 30 mm (9-F)
Qintar et al (2021)^5^	28, M	Acquired IVC thrombosis on background of MTHFR thrombophilia	RIJ for device delivery; left basilic vein for ICE	Steerable catheter	Agilis steerable catheter crossed PFO; occluder delivered directly through Agilis	Amplatzer PFO occluder 35 mm (10-F)
	24, F	Acquired iliac in-stent thrombosis (May-Thurner) with IVC clot burden	RIJ for device delivery; left basilic vein for ICE	Steerable catheter	Agilis steerable catheter, then TorqVue 120° sheath over Amplatz Extra Stiff wire in pulmonary vein	Amplatzer PFO occluder 35 mm (10-F)
Stehli et al (2019)^6^	73, M	Acquired thrombotic IVC occlusion post pulmonary embolism	Transhepatic after transjugular failure	Direct trajectory (transhepatic)	Transjugular attempt abandoned after recurrent wire prolapse; transhepatic access; wire escalated to Lunderquist Extra-Stiff for sheath delivery; complicated by hepatic artery injury requiring coiling	Amplatzer PFO occluder 25 mm (8-F)
Fanari et al (2016)^7^	78, M	Acquired thrombotic occlusion of IVC filter with bilateral DVT	Right internal jugular	Reshaped delivery sheath	8-F Amplatzer TorqueView delivery sheath manually reshaped into U-shaped curve; MP and CLS guides attempted initially	Multifenestrated ASD occluder 25 mm (8-F)
Węglarz et al (2014)^8^	26, M	Acquired thrombotic IVC occlusion (Crohn's, protein C deficiency)	Right internal jugular	LV wire anchoring (salvage)	JL 3.5 guide; Amplatz Ultra Stiff wire with manually preshaped tip advanced through mitral valve into LV after LA seating failed	Figulla Flex II PFO 23/25 mm (9-F)
Hascoët et al (2013)^9^	74, M	Acquired thrombosed IVC filter	Right internal jugular	Distal wire anchoring in pulmonary vein	Attain deflectable catheter to engage PFO; backup Meier wire seated in right upper pulmonary vein; 180° Amplatzer delivery sheath	Amplatzer Cribriform 25 mm (9-F)
Hussain et al (2010)^10^	67, M	Congenital IVC interruption	Transhepatic	Direct trajectory (transhepatic)	Hi-Torque Supra Core wire; 9 F Amplatzer sheath; Cook embolization coils for hepatic tract hemostasis	Amplatzer Cribriform 25 mm (9-F)
	76, F	Acquired thrombotic IVC occlusion; platypnea-orthodeoxia syndrome	Transhepatic	Direct trajectory (transhepatic)	Supra Core wire; 10-F Amplatzer sheath; Nester coil; transhepatic approach selected to avoid filter-displacement risk	Amplatzer Cribriform 25 mm (10-F)
Jolly et al (2010)^11^	71, F	Congenital atretic infrarenal IVC with secondary thrombotic occlusion	Transhepatic	Direct trajectory (transhepatic) with distal wire anchoring in LUPV	MP catheter; Amplatzer vascular plug for hepatic vein hemostasis (first reported use)	Amplatzer Cribriform 25 mm (8-F)
Carter and Cavendish (2008)^12^	52, F	Thrombosed IVC filter	Left axillary vein	Distal wire anchoring in LA	IMA catheter to engage PFO from SVC; stiff Amplatz wire with manually preformed curve looped in LA; 180° Amplatzer PDA delivery sheath	Amplatzer Cribriform 25 mm (6-F)
Sader et al (2003)^13^	40, F	Congenital infrarenal IVC occlusion	Right internal jugular	Distal wire anchoring in LA	Voda Left 3.5-mm guide to engage PFO from SVC; Amplatz J-wire looped in LA; 11-F Mullins sheath	CardioSEAL 23 mm (11-F)
	68, M	Surgical IVC ligation post DVT/PE	Right internal jugular	Distal wire anchoring in LA	Voda Left 3.5-mm guide; Amplatz J-wire looped in LA; redeployable Amplatzer device selected after CardioSEAL failed to seat	Amplatzer septal occluder 10 mm (11-F)

ASD = atrial septal defect; CLS = curved left support; DVT = deep vein thrombosis; EBU = extra back-up; ICE = intracardiac echocardiography; IMA = internal mammary artery; IVC = inferior vena cava; JL = Judkins left; LA = left atrium; LUPV = left upper pulmonary vein; LV = left ventricle; MP = Multipurpose; MTHFR = methylenetetrahydrofolate reductase; NR = not reported; PDA = patent ductus arteriosus; PE = pulmonary embolism; PFO = patent foramen ovale; RIJ = right internal jugular; SVC = superior vena cava.

The right internal jugular vein was the most common access used for device delivery (n = 9), followed by transhepatic (n = 5) and left axillary vein access (n = 1). Device types, imaging modalities, and sheath sizes are described in Table 1.

### Upper limb venous access

A single case used the left axillary vein for device delivery in a patient with an IVC filter and extensive iliocaval thrombus, arguing that this access provided a more favorable angle of approach than the transjugular access.^12^ Qintar et al^5^ used the left basilic vein for intracardiac echocardiography (ICE) catheter placement in both their transjugular cases, which avoided the need for general anesthesia and TEE. The capacity of the upper limb venous system for large-sheath delivery is less well-characterized than that for femoral or jugular routes although Qintar’s use of a 10-F sheath in the basilic vein without complications is informative.

### Transhepatic access

Transhepatic PFO intervention involves accessing the hepatic veins via percutaneous puncture through the hepatic parenchyma. Established in pediatric congenital interventions, transhepatic access approximates the transfemoral alignment between the IVC and the fossa ovalis, but delivery of large sheaths through hepatic parenchyma requires stiff support wires. Supra Core (Abbott), Amplatzer Superstiff (Abbott), and Lunderquist (Cook Medical) wires were used across the published cases,^6^^,^^10^ and at least one case used distal wire anchoring in a pulmonary vein.^11^ Complication rates in transhepatic cardiac access approach 5% in the largest published series, which is predominantly pediatric.^14^ Adult experience remains limited to small case series.^15^ In their transhepatic PFO closure, Stehli et al^6^ reported subcapsular hematoma and bleeding that required coil embolization of a hepatic artery branch and blood transfusion, underscoring the need for embolization coils, vascular plugs, or gelatin sponges for hepatic-tract hemostasis on sheath withdrawal.^2^^,^^6^^,^^10^^,^^11^ This approach is likely best suited to centers experienced in transhepatic interventions.

### Right internal jugular venous access

Approaching the PFO from the right internal jugular vein replaces the femoral approach’s favorable alignment between the IVC and the fossa ovalis with a more perpendicular angle. The passage of a wire and catheter from transjugular access requires a sharp transition from an inferior course through the superior vena cava (SVC) to a superior passage through the PFO into the LA, as demonstrated in Figure 3. This unfavorable crossing angle is the central technical challenge of the transjugular approach. The 9 transjugular cases in our literature search describe a range of techniques to overcome this obstacle. The procedure can be deconstructed into 2 sequential steps.

1. Engaging the PFO. The perpendicular and superiorly oriented path between the SVC and a PFO require the use of catheters that approximate this angle. This was achieved in 3 cases using steerable catheters that feature an operator-controlled deflectable tip which can be adjusted to achieve optimal PFO engagement.^5^^,^^9^ A curve too large or too small for the interatrial anatomy may require exchange for a catheter with a shorter or longer deflectable segment.^9^ Where preformed catheters were successful, they were predominantly left coronary catheters such as extra back-up,^3^^,^^4^ Voda Left,^13^ and Judkins left^8^ catheter shapes.
2. Crossing and sheath delivery. Once a catheter has engaged the PFO, a guidewire is advanced into the LA and used as a rail for delivery of a large (8-F to 11-F) delivery sheath through which the PFO occluder is deployed. Inadequate support at this stage will manifest in wire prolapse or failure of the sheath to track across the septum. Two broad strategies were employed to overcome this: changing the delivery sheath to improve engagement with the PFO, or distal anchoring of the wire beyond the septum to provide a stable rail. In the former case, the use of a large-bore steerable catheter can improve engagement with the foramen ovale and can also act as a delivery sheath for the PFO occluder.^5^ Manual reshaping of a TorqueView delivery sheath using warm saline has also been described as a means of addressing the more perpendicular angulation encountered from the transjugular approach.^7^

The left upper pulmonary vein is the conventional guidewire-anchoring position in transfemoral PFO closure, providing a straight rail through the IAS for equipment delivery. Despite being successful in 2 cases,^5^^,^^9^ the crossing angle from the transjugular approach means that pulmonary vein anchoring does not reliably provide the same mechanical support. Several of the published cases required techniques to augment that support or alternative anchoring positions. Ozdemir et al^3^ inflated a 5.0-mm coronary balloon over a 0.014-inch coronary guidewire in a pulmonary vein to facilitate delivery of an extra back-up guide which would not initially cross. Thereafter, they passed a curved stiff guidewire into the LA and used this anchor position to deliver the occluder device.^3^ Three further cases used looped 0.025-inch or 0.035-inch wires anchored in the LA to deliver PFO occluder devices.^4^^,^^13^ Weglarz et al^8^ reported directing an Amplatz Ultra Stiff Guidewire into the left ventricle after failing to obtain a stable anchor in the LA. This approach provides a straighter wire path than anchoring in pulmonary veins or the LA and most closely approximates the transfemoral approach.

Our case followed the two-step framework outlined earlier: A left coronary catheter (AL1) provided initial PFO engagement, and left ventricular wire anchoring provided the rail for sheath delivery once pulmonary vein anchoring proved inadequate. Weglarz et al^8^ previously described left ventricular wire anchoring as a salvage technique in this setting, using a manually preshaped Amplatz Ultra Stiff wire. Our case differs in that the Safari guidewire (Boston Scientific) is purpose-designed for stable, atraumatic positioning in the left ventricle during TAVR sheath delivery. Safari wire positioning in the left ventricle to support transjugular LA appendage occlusion has recently been reported, where the same SVC-to-septum angulation must be overcome.^16^ To our knowledge, ours is the first reported use of a dedicated preshaped TAVR wire for transjugular PFO closure.

## Future Directions

The level of evidence available is restricted to case reports, and no larger case series or prospective registry data exist. Alternative access for PFO closure is likely too rare a scenario for randomized evaluation. A prospective registry would allow a more reliable assessment of optimal techniques and outcomes. From this review, no single distal anchoring strategy emerges as mechanically superior. Case-specific anatomy and operator experience are likely to remain the major determinants of strategy selection pending further data.

## Conclusions

Percutaneous PFO closure in the setting of IVC interruption is technically feasible through transjugular, transhepatic, or upper limb venous access. This is the first dedicated review of alternative access approaches for PFO closure in this setting. The transjugular approach is the most frequently reported one and uses the access familiar to interventional cardiologists. The main technical challenge is the crossing angle between the SVC and the IAS. We present a two-step framework for PFO engagement and sheath delivery.

**Take-Home Messages**

• Transjugular and transhepatic access are both feasible alternatives for percutaneous patent foramen ovale closure in case of inferior vena cava interruption, with transjugular providing access more familiar to interventional cardiologists and avoiding the bleeding risks of a transhepatic puncture.
• The transjugular approach requires specific strategies to overcome the unfavorable crossing angle at the interatrial septum, including the use of preformed left coronary catheters, steerable catheters, and distal wire anchoring within the pulmonary veins, left atrium, or left ventricle.
• Where wire positioning in the pulmonary vein or left atrium offers insufficient support, placement of a preshaped transcatheter aortic valve replacement guidewire in the left ventricle is an additional option for establishing a rail across the septum.

## Funding Support and Author Disclosures

The authors have reported that they have no relationships relevant to the contents of this paper to disclose.

## Appendix

Video 1 Left Femoral Venogram Demonstrating Tortuous Collateral Venous System Around Occluded IVC and Reconstitution of the IVC at the Level of the Right Atrium

Video 2 Two-Dimensional TEE at 0° Demonstrating the Right and Left Atrial Disks of the 30-mm Amplatzer Talisman PFO Occluder Straddling the IAS Following Deployment
